# Repellents and New “Spaces of Concern” in Global Health

**DOI:** 10.1080/01459740.2017.1327957

**Published:** 2017-06-08

**Authors:** Ann H. Kelly, Hermione N. Boko Koudakossi, Sarah J. Moore

**Affiliations:** ^a^ Department of Global Health and Social Medicine, King’s College London, Strand, London, United Kingdom; ^b^ Department of Sociology and Anthropology, University of Parakou, Parakou, Benin; ^c^ Department of Epidemiology and Public Health, University of Basel, Basel, Switzerland; ^d^ Environmental Health and Ecological Sciences Thematic Group, Ifakara Health Institute, Bagamoyo, Tanzania; ^e^ Health Interventions Unit, Department of Epidemiology and Public Health, Swiss Tropical and Public Health Institute, Basel, Switzerland

**Keywords:** Domestic space, global health innovation, malaria, resistance, vector-control

## Abstract

Today, malaria prevention hinges upon two domestic interventions: insecticide-treated bed nets and indoor residual spraying. As mosquitoes grow resistant to these tools, however, novel approaches to vector control have become a priority area of malaria research and development. Spatial repellency, a volumetric mode of action that seeks to reduce disease transmission by creating an atmosphere inimical to mosquitoes, represents one way forward. Drawing from research that sought to develop new repellent chemicals in conversation with users from sub-Saharan Africa and the United States, we consider the implications of a non-insecticidal paradigm of vector control for how we understand the political ecology of malaria.

All spatial repellent systems attempt to prevent blood-feeding arthropods from reaching their target hosts within a space of concern.

– Kline and Strickman (2015:240)

The history of malaria control is littered with failed efforts to keep mosquitoes at bay. The malaria parasite is transmitted by mosquitoes belonging *Anopheles* genus, a vector remarkable in its highly selective preference for human blood and its fine-tuned capacity to seek out its favored host (Service 2008). Adapted to the vicissitudes of local conditions, Anophelines can diverge widely in behavior, leading to a dizzying array of sub-species and species complexes that defy taxonomic classification (Killeen et al. 2013). Co-evolving over millennia, humans, mosquitoes, and parasites are dynamically intertwined across diverse habits and habitats—a pathogenic multiplicity central to the anthropological concerns this anniversary issue seeks to explore (Chandler and Beisel, 2017).

In this article, we describe an entomological research project that takes the situational contingency of malaria as its starting point (cf. Kelly and Lezaun 2014). Malaria vector control, broadly considered, encompasses various techniques, and technologies designed to prevent infective mosquito bites. Current global health policy prioritizes indoor residual spraying (IRS) and long-lasting insecticidal nets (LLINs)—approaches that target the tendency of most *Anopheles* species to seek blood meals indoors. Coupled with rapid diagnostic tests and artemisinin-based combination therapies (ACT), these domestic strategies have made a considerable dent in malaria morbidity and mortality globally (WHO 2016). However, the selective pressures associated with large-scale IRS campaigns and mass ITN distribution programs have unfortunately worked to accelerate vector diversification, reducing the effectiveness of these frontline measures as an increasing number of species now tend to feed outside or are resistant to the insecticides used in these interventions (Russell et al. 2011).

To tackle these widening gaps in protective coverage, the WHO has endorsed a global program of Integrated Vector Management (IVM), resetting malaria control measures within a comprehensive initiative to address vector-borne diseases.^1^ Thoroughly eclectic in its approach, IVM champions site-specificity and health sector compatibility, matching vector control interventions—whether these involve environmental modification, spraying of chemical insecticides, household design, or (more recently) the release of genetically modified mosquitoes—to the distinct entomological, epidemiological, and administrative features of local contexts of transmission (e.g., Barik 2015; van den Berg 2009; Kirby et al. 2009; Touré and Knols 2006). IVM has also been positioned as an engine for disruptive innovation, bringing together profit and nonprofit actors to integrate markets for vector and pest control technologies (Knapp et al. 2015).^2^

The quintessential challenge of malaria vector research and development is how to achieve local epidemiological impact, while working at a speed and scale that can forestall the genetic and behavioral changes that targeted interventions will inevitably precipitate. Building bespoke and responsive vector control programs from intensive local entomological, epidemiological, and ecological surveillance is one way to ensure that the shifting vulnerabilities of mosquito populations remain squarely in the sights of malaria eradication. Spatial or area repellents constitute an original approach to the vision of an ever-more precise topology of global health research and action (cf. Kelly and Lezaun 2013). Spatial repellents diffuse through the air, driving vectors away before reaching their hosts. While technologies of repellency are certainly not new—Herodotus’ discussion of plant-burning to disperse biting flies is a popular reference in the literature—malaria research and development in this area have been limited (Norris and Coats 2017). The sheer insecticidal power of DDT has cast a long shadow over malaria research and development, orienting vector control interventions toward the killing of mosquitoes. This emphasis on the elimination of the vector, however, may not always be the most effective endpoint to reducing disease transmission (Roberts et al. 2000). With insecticide resistance on the rise, medical entomologists have called for “a paradigm shift from contact toxicity-based strategies to…modalities that can operate safely at a distance” (Achee et al. 2012:4).

In what follows, we explore the implications of a *distancing* strategy for how malaria is configured as a global health problem. Our analysis draws from our involvement in a Bill and Melinda Gates Foundation (BMGF) Global Health Project that evaluated novel spatial repellents for the purposes of malaria control. Tackling residual malaria is a central focus of the Foundation’s Global Malaria Action Plan. By extending protection over areas and populations outside the reach of current approaches, spatial repellents promise to advance global efforts to “Accelerate to Zero” and achieve global eradication of the disease (Strickman and Reddy 2016; White and Moore 2014).^3^ The global health potential of spatial repellents inheres not only in their anti-malarial efficacy but also in their unique economic value. The global mosquito repellent market is currently valued at 3.2 billion dollars and is predicted to reach 5 billion by 2022—a steady growth attributable both to emerging diseases and emerging markets.^4^ The sheer diversity of formats available—from topical creams, soaps, and sprays to vaporizing coils and candles, treated mats, and impregnated clothing—reflects the commercial value of repellents, often marketed alongside cleaning products, garden tools and camping equipment (Zhang et al. 2010). Our project’s central objective, therefore, was to not only to demonstrate that repellents, widely available for personal use, could have a considerable epidemiological impact, but also to explore how their global health applications could be underwritten by commercially driven innovation in the West. With the proper cross-sector engagement, vector-control repellents could come to represent the kind of marriage of humanitarian need and commercial investment driving much contemporary innovation in global health (McGoey 2015).

We begin by contextualizing the project’s attempt to refashion spatial repellents as global health tools within the contemporary history of malaria vector control. Of particular concern for our purposes is the continuing relevance of the household as the primary site for malaria control, and the spatial and social assumptions about domestic space that structure interventions. After a brief note on method, we draw together insights from fieldwork conducted with residents living in and near Cotonou, the economic capital of Benin, and two counties in Southwest Florida in the United States. This seemingly incongruous cross-weaving of sites speaks to the distinctive features of spatial repellents as “humanitarian goods” designed “to do well while doing good” (Redfield 2016; see also Beisel 2015; Street 2015). We then consider how mosquitoes are differently framed as a matter of concern when repellency is the preferred form of intervention and examine the relationships, expectations, and contestations that local responses to mosquitoes gather together (Latour 2004).

We conclude by reflecting on how this ethnographic exercise of working across diverse contents and contexts might alleviate the conceptual freight domestic spaces carry for global health. While the modern household is often understood as a site of separation (e.g., Douglas 1991; Sloterdijk 2004), vector-borne diseases brutally undercut the prophylactic capacities of the household and blur the boundaries between public and private spaces. Placing human–mosquito interactions within a “borderland of folded relations” (e.g., Hinchliffe and Ward 2014:143), we focus attention on the vicinities—the yard, the lawn, the threshold, back-alley plots, and pathways—that constitute malaria’s contemporary spaces of concern.

## Kill, nudge, and repel

The extraordinary successes of mid-century malaria control and its equally dramatic failures are largely due to one chemical compound: Dichloro-Diphenyl-Trichlororethane (DDT). Synthesized during the second world war, this “excellent powder” kills insects at low concentrations, is residually effective as an aerosol, and is cheap to manufacture (Roberts and Tren 2010). Applied on the walls of houses, DDT helped bring malaria transmission to a halt across Europe and North America (e.g., Harrison 1978; Russell 2001). Those successes were, however, tempered in sub-Saharan Africa. A combination of factors—for example, vector density, diversity, and resilience; climatic conditions favoring year-round transmission; political instability; and poor infrastructures—limited the impact of indoor residual spraying campaigns (Packard 2007).^5^ The collapse of the Global Malaria Control Program in the 1960s has now become a classic—if contentious—exemplar of the blind faith in a “magic bullet” technology, the neglected potentials of grassroots organization, and the hubris of scientists, policymakers and/or environmental activists (e.g., Carter 2007; Dunlap 1981; Kelly and Beisel 2011).

What is of little dispute is the speed with which mosquitoes acquired resistance to DDT.^6^ As the WHO reoriented its global malaria policy from eradication and elimination to local control, research came to focus on an alternative set of compounds (Litsios 1997). Pyrethroids—long-lasting chemicals that are highly toxic to insects but generally harmless to birds, humans, and other mammals—proved a highly effective front-line defense when applied to bed nets and have since been held responsible for the prevention of millions of malaria-related deaths (Greenwood 2008). That efficacy has sadly also proven finite: after almost twenty years using the same class of insecticides in the same way, mosquitoes are becoming increasingly resistant to pyrethroids throughout sub-Saharan Africa, Southeast Asia and elsewhere (Ranson et al. 2011; Chareonviriyaphap et al. 2013).

In developing the next generation of insecticides, the question of how to take into account the adaptive capacities of the vector is central (Ferguson et al. 2010; Takken and Knols 2009; WHO 2013). One option involves the rotation of formulations, including DDT. Cumulative insights on the impact of this chemical have shown that DDT’s toxicity to the mosquito is only one and, in terms of disease control, possibly the least effective of its chemical effects.^7^ In addition to killing mosquito on contact, the pesticide also works as a powerful repellent (Mabaso et al 2004; Ogoma et al. 2014; Roberts et al. 2000; WHO 1971).^8^

Repellency is a mode of action with manifold public health advantages. Preventing blood-seeking mosquitoes from entering a house or compelling them to leave early can indeed reduce vector survival rates through, for instance, starvation or exposure. But the effectiveness of repellents does not hinge upon vector mortality; mosquitoes that find it difficult to feed on humans may seek other hosts and, eventually, other places to breed (Syafruddin et al. 2014). If one considers that the dispersal and maturity of mosquitoes are directly related to their infectivity, the epidemiological impact of these subtle disruptions in vector bionomics is potentially far-reaching (Killeen and Smith 2007). Ultimately, these “vector free spaces” come by way of deterrence rather than elimination, a gradual “nudging” of mosquito behaviors that does not generate the selection pressures associated with the emergence of resistance (Choi et al. 2016).

Worldwide, the most common repellent is DEET (N.N.-diethyl-3-methylbenzamide). Like DDT, DEET was developed in the context of World War II to protect Allied forces deployed in the Pacific and North African theaters. Also tested for use in agriculture as a pesticide, DEET is highly effective against a number of biting insects and forms the basis of the majority of commercial repellents (Leal 2014). Yet for the purposes of disease control, DEET based products face considerable challenges. Because DEET is not a volatile compound, mosquitoes must come into direct contact or at the very least close proximity with a treated surface in order to be repelled. Moreover, while many formulations can last up to several hours, ultimately their potency depends upon reapplication. In tropical areas, where high levels of perspiration can shorten a product’s durability, adequate protection is costly and sometimes impracticable (Norris and Coates 2017).

In contrast, spatial repellents work beyond the surface of their application and generate atmospheric effects. This volumetric potential is critical for protecting against outdoor vector populations (Govella and Ferguson 2012; Durnez and Coosemans 2013). An area repellent radiates a protective atmosphere that insulates not only the individual within her discrete residence, but also nonusers living nearby. The range of protection will depend upon the properties of the chemical, its application platform, and environmental conditions such as airflow, temperature, and humidity. But across formulation and format, these chemical “bubbles” do not require physical contact with mosquitoes and can thus afford a more robust protection against multiple malarial situations (Grieco et al. 2007). Furthermore, they gain additional relevance in the context of rapid urbanization across the tropics, specifically for the large proportion of the population living in slums, as vector-human contacts become increasingly difficult to contain within four walls (Reddy et al. 2011).

While the epidemiological case for dislocating control from domestic surfaces is compelling, what is known about infectious disease does not necessarily tally with what is feasible. The household remains a powerful unit of public health policy and intervention, upon which malaria control and global infectious disease control initiatives continue to rely (see Jerven 2013). Defining the appropriate sociopolitical register for a program of prevention that operates in the spaces *between* residences remains a critical question. Thus, in an effort to support the development of a sub-lethal vector control paradigm, we directed our anthropological attention to the everyday cleavages in domestic, public, and natural space and the opportunities for distancing humans and mosquitoes these lived domestic landscapes opened up.

## Methodologically adjacent

Anthropological insights into mosquitoes have generally served to situate vector control programs, elucidating how local understandings of the disturbance mosquitoes pose can help refine efforts to reduce disease transmission (e.g., Okrah et al. 2002; Winch 1999). Putting ethnographic depth at the service of infectious disease control is a notoriously tricky undertaking—a balance that requires attending to the political implications and limitations of public health engagement more broadly (e.g., Marsland 2006; Montgomery, Munguambe, and Pool 2010; Nading 2012; Panter-Brick et al. 2006). Our collaboration takes its cue from this scholarship, seeking to understand better how people experience mosquitoes, and bringing that interspecies attention to bear on the design of new global health interventions. Our agenda, however, departs from other anthropologies of vector control in two important ways. First, the repellent platform investigation was still in development at the time of our research. Different delivery formats—for instance, emanators, candles, and coils—had been prototyped and trialed, but just which of these should be scaled up into a malaria control intervention was one of the questions the project sought to address (Ogoma, Moore, and Maia 2012). The open-endedness of the intervention shifted the nature of our ethnographic work from evaluative to imaginative and offered our interlocutors the opportunity to formulate solutions to vector-control based on their own diagnosis of the problem (c.f. Otto and Smith 2013).

Second, the process of product development was here constrained by the need to address highly diverse different markets. That goal introduced a degree of multi-sightedness to the ethnographic work that may seem at odds with how anthropological work in global health has commonly been understood. We conducted fieldwork in two contexts: 1) the periurban areas around Cotonou, Benin, where malaria is the leading cause of death for children under five and insecticide resistance is on the rise; and 2) Florida’s Manatee and Monroe Counties, which over the past five years have witnessed the ramping up of mosquito control programs to combat Dengue and West Nile Virus, but where the risk of mosquito-borne disease remains comparatively low. In each setting, we used a mixed method approach that combined survey data, interviews, focus group discussions, and ethnographic fieldwork in order to explore the situated experiences of risk vis-à-vis the array of protective measures taken against mosquitoes. Our aim was not to represent, let alone compare, “developing” or “developing world” communities. Rather, our empirical work served as a scoping exercise to identify public health practices that could run across radically distinct sociopolitical contexts. This remit allowed for considerable investigative latitude.

In Benin, research began with a structured questionnaire. Our hope was to outline the socio-demographic characteristics of the area and the various local approaches to mosquito control. Three townships around the Lagoon of Contonou—Ladji, Abomey-Calavi and Akron—were selected for their ethnic diversity and shared vector ecology. In all of these areas, insecticide resistance is on the rise. Households were randomly sampled based on data collected by the Center of Entomological Research of Cotonou (CCREC), the arm of the Ministry of Health in charge of national malaria control activities. Overall, 250 households were approached: 50 in Akron, 75 in Ladji, and 125 in Abomey-Calavi, in proportion to the size of the township. The data were analyzed with the Statistical Package for Social Sciences (SPSS) 13.0; key parameters were described by simple proportions. Fifty households presenting relevant characteristics—for example, those that had at least two children living at home, or families that spent either a great deal or relatively little money on control methods—were selected for follow-up interviews. Qualitative observations were focused on the social, material, and temporal configuration of the household, and on characterizing the points at which mosquitoes surfaced as a problem within the everyday practices of domestic living.

In Florida, where until recently vector-borne diseases had rarely been a cause for community-level action, our research began with County Mosquito Control Departments. While the problems mosquitoes pose for Monroe (which includes Key West) and Manatee Counties are roughly similar, at the time of the study the former’s budget for mosquito control outstripped the latter by roughly 12 million dollars. Of particular, interest, then, was how individual’s efforts to protect against mosquito bites tallied with what Robbins, Farnsworth, and Jones have called the “jurisdictionally bound geographies of responsibility” (2008:14). Taking inspiration from their work on mosquito management in Arizona, we followed the patterns of residential variance in mosquito control, conducting focus group discussions with members of Home Owner Associations (or gated communities), nursing homes and in mobile trailer parks (eight in all). Domestic inspectors in Manatee and Monroe Counties provided the platform for ethnographic work, which involved daily door-to-door checks to locate and in some instances treat mosquito breeding grounds. Follow-up interviews were conducted with fifteen residents who were struggling to control mosquito populations in their yards (Cann 2012).

Finally, an online survey (n = 181) was circulated through emails collected during “health fairs”—short educational forums periodically organized by the Department of Health in collaboration with community based organizations and the University of Miami. These sessions, which provided an important context for health care outreach and delivery, offered an occasion to extend and diversify the participant base of our study. The survey questions were designed in collaboration with mosquito control officers and focused on respondents’ understandings of vector-borne disease, preferred modes of protection and views on the role of public authorities in mosquito control. Online survey responses can be difficult to validate and are representative only of the kinds of people who might attend the “health fair” and take the time to fill out a survey. Integrated with ethnographic accounts, focus group, and interview data, however, these survey-based assessments of mosquito control practices and preferences deepened our insights into the kind of repellency formats that might be worth developing for explicitly public health purposes in these contexts.^9^

## The Lagoon of Cotonou

In the three coastal townships included in our study, over 80 percent of survey respondents were tenants, typically renting a single unit within a three or four-room structure. These properties do not constitute a sealed domain; valuables or food stores might be covered with a plywood or aluminum sheeting, while others might be left open to the air. During the dry months, people tend to sleep outside, stretching plastic sheets over a floor area for protection when the rains begin. Despite public ordinances restricting the number of residents per room to no more than three, houses are generally overcrowded, often accommodating large extended families.

In Abomey-Calavi, which is near the university and where rents tend to be higher, bathrooms are often built in the back of the house and connected to a shared septic pit. In contrast, most of those living in Ladgi and Akkron use a hand-dug toilet in the yard (c.f. Jenkins and Curtis 2005). Across all three areas, however, domestic life was primarily conducted out of doors: residents cooked, slept, and bathed outside, making use of communal infrastructures and free-standing walls. “I rent this home,” one mother in Ladgi commented:

It is just here, we keep our mattress, our food and clothes, here inside. There is no room for cooking, this is done here in front. At night, the baby sleeps with me on the mattress under the net, but we are many, and it is hot. The older ones will sleep outdoors on the matt.

The vast majority of those surveyed (88 percent) regarded mosquitoes as a public health problem; roughly two-thirds (63 percent) owned and used insecticide-treated bed nets (ITNs) to protect themselves from malaria. With the support of the US Presidential Malaria Initiative, authorities in Benin have carried out a systematic distribution of impregnated mosquito nets at the beginning of the raining season and, since 2011, have introduced indoor residual spraying (IRS) into areas determined to be at particularly high-risk, including those located around the Lagoon of Cotonou.^10^

That being said, only 22 percent of survey respondents claimed to use ITNs all year long, with most people limiting their use to the rainy season, and even then, just to protect their children. In interviews, residents reported refashioning freely provided nets into sponges, car covers, or fishing nets. Few respondents (9 percent) made the association between the presence of mosquitoes and environmental care, such as dumping standing water or putting lids over toilets. While malaria was understood to be a feature of everyday life—“it is a fever we all have, this is something we do not escape”—mosquitoes’ role in disease transmission was thought to be circumscribed to the night-time, when people thought they were most vulnerable to bites.

In light of successive national bed-net education campaigns, the association between sleeping and mosquito infectivity is understandable. Moreover, because ITNs were freely distributed, people tended to link malaria control to either broader governmental initiatives or the charitable work of NGOs enlisted by the state to support malaria prevention efforts. When it came to managing mosquito nuisance, however, residents proactively deployed a range of products—for example, smoke and coils, powders bought from Chinese sellers, insecticides sprays such as Baygon®, burning, and burying a local plants (i.e., *Citrus sinensis; Azadirachta indica; Hyptis suaveolens*). A strong preference was expressed for products that “smelled nice” or rather did not smell “too strong,” which was seen to interfere with cooking. The financial impact of repellents was often remarked upon. Women, who by and large took up responsibility for managing the domestic environment, complained that a significant portion of their annual household budget went into purchasing these products.

While topical formulations were occasionally used, applying chemicals to skin generated some anxiety. “I cannot escape the smell,” “this is not for my child,” and “we cannot put this on all the time” were common responses to questions about the use of repellent creams. During interviews, residents also expressed concerns about indoor residual spraying (IRS), and more than one occasion raised questions of the relationship between the chemical sprays and infertility (c.f. Montgomery, Munguambe, and Pool 2010). These expressions of distrust were largely directed toward government, whose attempts to carry out broader improvements in sanitation and flood protection were regarded with skepticism. In the wake of accusations that implicated the president in the collapse of a US $347 Ponzi scheme, fleecing hundreds of thousands of people of their life savings, the discrepancy between taxes and visible improvements to living conditions was frequently noted: “we don’t see much” was a leitmotif in interviews. The government was regarded as removed from efforts to achieve the “good life”—a project that depended upon personal initiative with the support of local social networks (c.f. Jenkins and Curtis 2005).

## Wetlands and swimming pools

In South Florida, mosquitoes are also generally accepted as an inescapable nuisance. However, the specter of persistent and emerging vector-borne diseases like Dengue Fever, West Nile Virus, St Louis Encephalitis, Chikungunya and, most recently, Zika, has raised the public profile of mosquitoes as a health problem.^11^ The CDC’s “Fight the Bite” campaign, launched in 2008, responded to those concerns by building awareness about strategies to reduce vector contact. Sensitization materials and messages stress the combination of individual precautions against bites—the use of sprays and creams, dressing in long pants in the evenings—and preemptive measures against mosquito breeding through property management. The latter has become a salient issue in Florida, which consistently sows the highest foreclosure rate in the US. In Manatee and Monroe Counties, where many sub-prime mortgages were taken out by “snowbirds” to purchase second homes, vacant properties, overgrown yards, and abandoned swimming pools have become prolific breeding grounds. Public health officials, moreover, are barred from areas that have been taken over by a bank. One health inspector explained:

The bank isn’t going to take responsibility for the public health of the local people by maintaining the property until it is resold, and if we need to get onto the property to treat a septic tank or take a look at the pool for breeding, we are going to have to find a way to get in.

Perhaps the most striking difference between Manatee County and Monroe Country is in their density of their population centers. The properties in Manatee County had much bigger yards and front lawns, and the houses themselves seemed to appear larger from the outside. In these areas, residents tended to blame neighbors who failed to tend their laws and clean their swimming pools. Residents of housing associations, or gated communities, identified immigrant communities as the source of the problem; “new” and “probably illegal” arrivals were deemed incapable of abiding by property laws and were potential carriers of disease agents. The threat immigrants posed to public health was framed as part of a larger failure of government regulation, which could also be seen in the lack of surveillance of spaces between private residences, described as “no-man’s lands.”

By way of contrast, in Monroe Country, which is largely composed of the small island of Key West, space is at a premium. With houses built closely together, the collective dimensions of mosquito control are amplified. As one inspector bemoaned, “a single resident can be responsible for mosquito problems on the entire block.” Renowned for their open and relaxed lifestyle, Monroe residents expressed the importance of individual preference when it came to lawn maintenance and the invasive character of vector surveillance: “How can you tell people that they are free to be who they want and do what they want, ‘but you must dump your standing water’, that just doesn’t sit right.” In deference to the “come as you are” ethos that makes the island attractive to tourists, the Monroe Public Health Department has gone to considerable lengths to train its inspectors on the protocols of gaining consent before entering a property. In contrast to the strict regulations for dumping standing water issued in Manatee, vector-control in Monroe tends to involve large-scale areal spray or fogging campaigns. While generally appreciative of the level of public health activity, Monroe’s residents also expressed a degree of skepticism of the effectiveness of these measures and considerable concerns about their environmental implications (c.f. Robbins, Farnsworth, and Jones 2008).

Survey data and focus group discussions indicated that a broad range of commercial repellants and insecticidal sprays was used to protect against mosquito bites in South and Western Florida, although these products were regarded with some ambivalence. As one elderly resident in a nursing home commented: “Well, it can’t be too healthy to breathe in the fumes from the spray, especially when I already have asthma; I think I would be worried it would trigger something. So I just stay indoors in the early morning and early evening.” Many expressed a preference for “naturalistic” methods, including wearing long pants, eating garlic, and squirting water mixed with washing-up liquid in areas where mosquitoes were believed to breed. Anything more “chemical” was perceived to be potentially damaging to the lawns and to emit odors that might interfere with outdoor activities in the evening. Concerns about the toxicity of repellents were particularly strong among parents with young children. “Do you know what I can use to put on my daughter?”, a mother asked a mosquito inspector making his rounds:

She is eighteen months old and wakes up in the morning with sometimes six to seven bites all over her body. It’s horrible for her, and it’s horrible for me to see her like that. I don’t want to put DEET on her, her skin is sensitive, but at the same time, it really bothers me that she has these bites. I do have screens on the house, you know, on doors and the windows, but somehow they still seem to get in.

The tension between avoiding mosquito bites and being exposed to potentially toxic chemicals ran through discussions and interviews. Moreover, while cases of dengue fever, for instance, were shared as cautionary tales, the risk of contracting vector-borne disease was generally discussed in the abstract. Residents of both communities articulated the presence of vectors as part of the sociopolitical climate of contemporary domestic life—each bite was a testament to the presence of illegal immigrants, economic downturn, or the inadequacy of governmental action.

## Global health vicinities

The differences between these peridomestic settings are striking. In terms of health risk, hand-dug latrines and annual flooding are clearly of a different order than abandoned swimming pools and ill-kept lawns. But in juxtaposing the ways in which communities in Benin and Florida frame mosquitoes as a matter of concern, some common features of these residential landscapes are brought into view. From the Béninoise *taudis* (slums) to Floridian vacation homes, domestic space is experienced as a frontier, a dynamic set of associations between human and nonhuman others, governmental directives, investments, and contaminants (cf. Reno 2011). The social and political polyvalence of the peridomestic, moreover, is reflected in the ways in which people understood their responsibility for mosquito control. In Benin, being at risk for malaria is associated with the vulnerabilities of sleep and thus is best managed through the use of bed nets, procured by the government and distributed by NGOs. These steps were held as somewhat distinct from the efforts to reduce the daily irritation mosquitoes posed. Reducing infiltration of buzzing and biting insects was taken up as a part of household management, driven by consumer choice, with strong preferences for products whose smells do not interfere with cooking.

In Florida, the recent influx of “tropical” diseases has brought the spotlight on mosquitoes as a public health concern, shifting attention to areas beyond property lines and exacerbating fears about the shifting demographic profiles of neighborhoods. Discussions about vector control become an opportunity to criticize a federal government deemed to be too lax on immigration, or local institutions failing at their duties to maintain green spaces and public lands. At the same time, residents feel conflicted about the extension of the state into private property, particularly when such responses involve restrictive ordinances or blanket applications of pesticides. A turbulent real estate economy accentuates the salience of peridomestic beatification; yards landscaped in a “tropical style” can help secure property values. In his study of American suburban ecologies, Paul Robbins (2012) has examined the ways in which lawn care, a practice of intensive social regulation, can be productive of communities through a shared sense of responsibility. “A neighborly concern,” maintenance of outdoor environments through the application of pesticides, herbicides, and fertilizers, is highly fraught (Robbins 2012). While essential to sustaining the collective aesthetic of community life, the volatile ecology of treated lawns raises troubling questions about the trade-offs among individual health, consumer preference, public responsibility, and environmental risk.

The perceived toxicity of chemical sprays also featured in discussions with Benin residents. Fear of infertility through insecticide use is widespread through Africa (e.g., Kaufman et al. 2012; Whyte, Van der Geest, and Hardon 2002), a legacy of colonialism replayed within the context of contemporary global health research (cf. Fairhead, Leach, and Small 2006). To garner local support, government-led and internationally supported IRS spraying initiatives have engaged in mass education campaigns and capacity-building initiatives to increase community engagement with vector control and entomological monitoring (cf. Chaki et al. 2011). It is unclear how these IRS campaigns will be taken forward by the National Malaria Control Program once the US Presidential Malaria Initiative steps back from operational supervision. Arguably, spatial repellents could help bridge emerging gaps in coverage by offering a cheap, relatively nonintrusive tool for which local communities could exercise some ownership. However, much depends upon the ways in which these tools are marketed. Protection against a disease risk perceived as negligible or even unimaginable could delimit public uptake or worse and play into latent fears that these chemicals are being introduced for nefarious reasons.

Greater consideration of the resonances between these tropical vicinities could also help enrich global health practices across diverse public health settings. A product of colonial intervention, the so-called African “household,” often sits awkwardly with public health interventions (e.g., Bonneuil 1999; Comaroff and Comaroff 1992; Guyer 1981). Polygamous practices, dense kinship networks, shared, and dynamic living spaces tend to elude economic and demographic categorization. Early twentieth-century insect campaigns were often leveled at the scale of the city or rural landscapes, in the effort to make these spaces more inhabitable to white settlers or amenable to agricultural cultivation (Caprotti 2006). While more recent public health interventions have emphasized the importance of involving local communities, the pragmatics of public action remain under-theorized, betraying a reified understanding of political identify at odds with the social fluidity that characterizes urban life in African cities (see Simone 2004).

The history of pest control in Europe and the Americas provides a comparably fertile ground to question the scalar demarcations of the private sphere that underpin public policy (e.g., Humphreys 2001). In her historical analysis of pesticides in US public housing around the time of World War II, Biehler (2009) notes how the discovery of DDT shifted policies from community management and quality building design to the personal maintenance of individual units. While this “bounding” of dwellings allowed for a more efficient control system based on private contracting of exterminators, in neglecting the spaces between apartments, it also prompted more tenacious infestations of co-habiting insects:

The poisons that were supposed to serve as chemical barriers in individual units actually repelled pests into the gaps between apartments, where they could breed freely and re-enter human living spaces. Cockroaches defied individualized approaches to pest control in public housing, taking advantage of dwelling units’ permeability (Biehler 2009:1020).

Recent global health policy attention to housing quality presents a latter-day iteration of the limitations of the domestic unit for improving population well-being (WHO 2011). Efforts to quantify and compare the health impacts of different types of construction and building materials have focused on degrees of moisture, temperature, ventilation, and exposure to pollutants (Haines et al. 2013). In these analyses, health and disease risk are constituted through the ambient conditions of domestic atmosphere—a fluctuating ecology of human and nonhuman biologies, material qualities, and processes (Garnett 2017).

That houses are entities in time as much as is place is an anthropological truism. As Mary Douglas (1991) suggested, rather than representing an impermeable material surrounding or a fixed inside-outside boundary, homes provide an orientation and coherence to the life of its inhabitants. While a home need not be fixed in space, it does presume a degree of regularity, a mode of control that arises out of shared project of co-existence. “Every service and transfer is part of an ongoing comprehensive system of exchanges, within and between the generations,” Douglas argues. “The most subversive attack on the home is to be present physically without joining in its multiple coordinations” (1991:302).

Closer attention to those domestic coordinations that mediate and produce pathogenic situations opens new avenues for vector-control (cf. Nading 2017; Shaw, Robbins, and Johns 2010). Proliferating in gutters and back alleys, in the seams that run between curbs and walls, mosquitoes problematize notions of the stable, bounded, private home. Domestic space as a protective structure is central to the modern ethos: Peter Sloterdijk (2004) likens modern domestic architecture to “a spatial immune system.” “Residence” he argues “is, immunologically speaking, a defensive measure designed to demarcate a sphere of well-being from invaders and other agents of unwellness” (Sloterdijk 2004:535). Yet the effort to establish a defendable border, “sealing off” the inside from a threatening exterior, clearly does not tally with the realities of everyday life or vector-borne infection.

“The ultimate goal of spatial repellents,” Debboun and colleagues write, “is to create a vector-free space. That space could be a picnic area occupied for a couple of hours a rural hut occupied every night. The space might also be mobile, such as a car or a theoretical bubble surrounding a hiker” (2014:245). With the threat of emerging and re-emerging diseases, we confront broad-based problems regarding the appropriate social, political, and spatial orders of control. Further sustained attention to the vicinities of transmission risk might prompt some new ways of imagining protection. By generating atmospheres distinct from the physical demarcations of the interior, spatial repellents offer a different kind of immunological vision, one associated with modularity, co-fragility, and reciprocal isolations.

## Notes

^1.^ See WHO/Vector Control Advisory Group (VCAG) on new tools. Geneva, Switzerland: World Health Organization. http://www.who.int/neglected_diseases/vector_ ecology/VCAG/en/

^2.^ The proliferation of product development partnerships characterizes Global Health R&D. In contrast to research in neglected diseases, however, where public-private-partnerships address situations of underfunding or “market failure” (Moran et al. 2010), innovations in vector control has long been a priority area for state-sponsored military and agricultural research in the West. The “newness” of these partnerships per se is therefore less remarkable than the changing ways in which government-industry collaborations are currently integrated into a global health agenda.

^3.^ D. Strickman and M. Reddy, Malaria Vector Control Research Agenda. Presentation to the Bill and Melinda Gates Foundation, February 3, 2016, see also http://blog.theglobalfight.org/malaria-vector-controls/.

^4.^ For a more detailed market breakdown, see: https://globenewswire.com/news-release/2017/02/07/914448/0/en/Global-Mosquito-Repellent-Market-will-reach-USD-5-00-Billion-by-2022-Zion-Market-Research.html

^5.^ These difficulties with malaria eradication were anticipated. Despite having the gravest disease burden, Africa was never fully included in the Global Malaria Eradication Program (Dobson et al. 2000).

^6.^ Resistance encompasses multiple different pharmacokinetic interactions from modifications in mosquito’s metabolism to structural changes in the insect’s exoskeleton to behavioral changes such as feeding outdoors.

^7.^ While early entomological studies had explored how exactly DDT worked, the “shoot first ask questions later” mode of the first malaria eradication campaign left little room to translate nuanced evidence of mosquito behaviors into policy (e.g. Smith and Webley 1969). The absence of entomologists on malaria policy—including the Vannevar Bush Insect Control Committee—is a testament to marginalization of any knowledge that might slow down the eradication agenda (Russell 2001).

^8.^ Pyrethroids have also been shown to have both toxic and repellent effects, raising questions as to which characteristics of the vector population—e.g. density, distribution and fecundity—are the most important parameters in malaria transmission.

^9.^ We used “smartsurvey”, a free online platform. The survey included 22 questions with a mix of single-answer, multiple answer, and Likert scales, designed to take roughly 5 minutes. See: http;//www.smartsurvey.co.uk/s/36647itkbn. The survey was sent out to 500 participants, with a response rate of 36.2.

^10.^ See the President’s Malaria Imitative, Benin, 2011. https://www.pmi.gov/docs/default-source/default-document-library/malaria-operational-plans/fy11/benin_mop-fy11.pdf?sfvrsn=6

^11.^ On one Friday in July 2014, for instance, 240 calls per hour, or one every minute, were logged in the Miami-Dade country office, complaining about the presence of mosquitoes and triggering an aerial spray campaign.
